# Motor Skills, Heart Rate Variability, and Arterial Stiffness in Children with Autism Spectrum Disorder

**DOI:** 10.3390/healthcare11131898

**Published:** 2023-06-30

**Authors:** Luděk Kalfiřt, Chia-Ting Su, Chung-Pei Fu, Shin-Da Lee, Ai-Lun Yang

**Affiliations:** 1 Institute of Sports Sciences, University of Taipei, Taipei 11153, Taiwan; d10831008@go.utaipei.edu.tw; 2 School of Culinary Arts and Hospitality Management, Na Celně 281, 29301 Mladá Boleslav, Czech Republic; 3 Department of Occupational Therapy, College of Medicine, Fu Jen Catholic University, New Taipei City 24205, Taiwan; 078566@mail.fju.edu.tw (C.-T.S.); 080718@mail.fju.edu.tw (C.-P.F.); 4 PhD Program in Healthcare Science, Department of Physical Therapy, China Medical University, Taichung 406040, Taiwan; 5 School of Rehabilitation Medicine, Weifang Medical University, Weifang 261000, China

**Keywords:** autism spectrum disorder, child, motor skills, heart rate variability, arterial stiffness

## Abstract

The prevalence of autism spectrum disorder (ASD) among children has been recently increasing. The severity of symptoms greatly varies between individuals with ASD, ranging from relatively mild to extremely severe. It is important to have a clearer understanding of the possible adverse consequences resulting from this disorder, such as delayed motor development, autonomic dysregulation, and arterial stiffness. Thus, the objective of this study was to investigate differences in motor skills, heart rate variability (HRV), and arterial stiffness between children with ASD and typically developing children. In this study, the school-aged children with mild symptoms of ASD (*n* = 17, 11.1 ± 1.0 years old) and typically developing peers (*n* = 15, 11.0 ± 0.5 years old) were recruited. Motor skills, HRV, and arterial stiffness were measured in these two groups. Motor skills were evaluated by the Bruininks–Oseretsky Test of Motor Proficiency-Second Edition. Moreover, HRV was measured through a short-term recording using the Polar heart rate monitor, and arterial stiffness was assessed by non-invasive computerized oscillometry. Compared with the typically developing group, children with ASD displayed significant deficits in some areas of motor skills, including manual coordination, strength and agility, and total motor composite. Moreover, children with ASD exhibited significantly reduced HRV, including time- and frequency-domain measures. However, the results did not demonstrate any statistically significant differences in arterial stiffness between the groups. Our findings demonstrated the presence of motor skill deficits and autonomic dysregulation in children with ASD.

## 1. Introduction

Autism spectrum disorder (ASD) is defined by the American Psychiatric Association as “a complex developmental condition involving persistent challenges with social communication, restricted interests, and repetitive behavior” [1]. The prevalence of children diagnosed with ASD has been steadily increasing in recent years. In 2018, it was estimated that the prevalence of ASD among American children was 1 in 44 children compared to an estimation of 1 in 54 children from 2016 [2,3]. It has been previously suggested that children with ASD experience delayed motor development [4,5]. Moreover, children with ASD tend to spend more time enacting sedentary behavior compared to their typically developing (TD) peers [6]. It is likely that a low level of motor skills negatively influences the physical activity participation of children with ASD [7]. In addition, delayed motor development in children with ASD negatively affects the overall health-related quality of life of these children [8]. Delayed motor development might be exacerbated in children experiencing more severe symptoms of ASD. In particular, children affected by non-verbal ASD might experience additional obstacles in motor development due to the manifestation of severe symptoms and its related difficulties with socialization. Even though the majority of previous studies have supported the presence of motor skill deficits in children with ASD, inconsistent findings regarding deficits in specific motor domains have been reported [4,5,9]. Therefore, it is important to have a clearer understanding of specific motor domains in which children with ASD underperform in order to design suitable and effective motor interventions.

Restrictive repetitive behavior, which is a key criterion for the diagnosis of ASD, has been previously linked to altered autonomic nervous system (ANS) functioning in children with ASD [1,10]. Heart rate variability (HRV) is a frequently used measuring tool for evaluating the function of ANS. It is indicated by the changes in the time intervals between consecutive heartbeats [11]. While increased HRV is associated with a variety of positive health outcomes, reduced HRV is connected to adverse health outcomes, such as chronic inflammation or cardiac dysfunction [12]. A limited number of previous studies have suggested that children with ASD exhibit reduced HRV compared to TD children [13,14]. Reduced HRV in combination with insufficient physical activity in children with ASD might likely lead to adverse health consequences later in life [6,13,14]. Therefore, it is crucial to provide a sufficient amount of evidence about potential ANS dysregulation in children with ASD.

Increased arterial stiffness, which refers to the rigidity of the arterial wall, is strongly associated with the higher risk of cardiovascular disease (CVD) [15]. A previously published systematic review demonstrated a significant association of higher body mass index (BMI) and lower cardiorespiratory fitness with increased arterial stiffness in TD children [16]. Similarly, a positive association between BMI percentile and arterial stiffness has been demonstrated in children with ASD [17]. Unfortunately, it has been shown that children with ASD display higher BMI and possess lower cardiorespiratory fitness levels in comparison with their TD counterparts [18]. For this reason, it seems that children with ASD might be at a greater risk of arterial stiffening and developing CVD later in life. However, only a single study examining arterial stiffness in children with ASD has been published to date; therefore, more evidence is urgently needed [17]. The objective of the current study was to investigate differences in motor skills, HRV, and arterial stiffness between children with ASD and TD peers.

## 2. Materials and Methods

### 2.1. Participants

A total of 32 participants were recruited in this study. All of the participants were school-aged boys from Taiwan. The age of the participants ranged from 9 to 13 years. The participants in the current study were divided into either the ASD group (*n* = 17, 11.1 ± 1.0 years old) or the control (typically developing) group (*n* = 15, 11.0 ± 0.5 years old). Participants allocated to the ASD group were diagnosed with ASD by a medical professional [1]. Regarding the extent of ASD, all the children with ASD were recruited from regular classes in public schools, where children with more than mild symptoms of ASD were excluded and referred to special classes or schools. In addition, these children had to exhibit typical IQ range and no comorbidities. On the other hand, participants allocated to the control group did not have a formal diagnosis of ASD or any other disabilities, which was confirmed by their school teachers and a medical professional. The current study was approved by the Institutional Review Board of Fu Jen Catholic University, Taiwan (C106058, 20 April 2018). All the participants and their parents provided a written informed consent before the start of experimental procedures. Basic anthropometric parameters, including body weight and height, were collected from the participants. The body weight of the participants was assessed by digital scale and height was measured using a portable stadiometer. In addition, body mass index (BMI) was calculated as weight in kilograms divided by height in meters squared.

### 2.2. Motor Skills Assessment

The Bruininks–Oseretsky Test of Motor Proficiency-Second Edition (BOT-2) was used to evaluate motor skills of the participants. The BOT-2 is a standardized motor assessment tool used for the evaluation of gross and fine motor skills in individuals between the ages of 4 to 21 years. The BOT-2 is intended to be used for the identification of potential motor delays, evaluation of the effectiveness of motor interventions, and as the support for the diagnosis of motor impairments. In the present study, motor skills of the participants were evaluated using the complete form of the BOT-2 according to the test manual, and the BOT-2 was administered to the participants in the school setting. The BOT-2 evaluates performance in four motor composites, each motor composite is divided into two subtests and each subtest consists of five to nine test items. Motor composites consist of fine manual control, manual coordination, body coordination, and strength and agility. Furthermore, motor composites include eight motor subtests: fine motor precision, fine motor integration, manual dexterity, upper-limb coordination, bilateral coordination, balance, running speed and agility, and strength. In total, the BOT-2 consists of 53 test items. The administration of the BOT-2 for a single participant requires from 40 to 60 min. The results of motor composites are calculated from sex- and age-specific standard subscale scores. In addition, total motor composite is generated by adding up the scores of four motor composites. The results of subtests are reported in scale scores, which represent results of the tested sample compared to normative data of the age-matched individuals of the same sex. The results of motor composites can be expressed as standard scores [19]. The BOT-2 has been frequently used for an assessment of motor skills and as a screening tool for potential motor deficits in the youth with ASD [20]. In addition, it has been demonstrated that the BOT-2 presents a good internal consistency for an evaluation of motor skills in the youth with ASD [20]. Furthermore, Downs et al. reported that the BOT-2 demonstrates the highest level of validity and reliability when used as an assessment of children with ASD in comparison with other commonly used motor assessment tools [20]. Moreover, Downs et al. classified the feasibility of the BOT-2 when assessing children with ASD as satisfactory, with the components of time, space, and training requirements scored as fair and the component of equipment requirement scored as good [20]. Lastly, Downs et al. concluded that the BOT-2 is the most psychometrically appropriate motor assessment tool for children with ASD [20].

### 2.3. Measurement of Heart Rate Variability

The HRV of the participants was indicated via the measurement of a short-term HRV using the Polar heart rate monitor (RS800CX, Kempele, Finland). HRV data were detected by a 5-min recording at rest, with the participants maintaining a supine position in a quiet, private, and semi-darkened room. Throughout the measurements, ambient room temperature was maintained between 22 and 24 °C. The instructions to avoid caffeine and any intense physical activities for 24 h before the assessment of HRV were given to the participants. Furthermore, all the measurements were conducted before noon. The HRV analysis software (Nevrokard, Izola, Slovenia) was applied for analyzing the time- and frequency-domain measures. Mean heart rate (HR), mean normal-to-normal intervals (NN), standard deviation of the NN intervals (SDNN), and root mean square of successive NN interval differences (RMSSD) were included in the time-domain measures. The low-frequency (LF) power (0.04–0.15 Hz), high-frequency (HF) power (0.15–0.4 Hz), and total power, and the ratio of LF-to-HF power (LF/HF) were included in the frequency-domain measures. Except for LF/HF, LF and HF were log transformed to account for unequal variances and to improve interpretability of the data. In this study, Poincaré plot standard deviation perpendicular to the line of identity (SD1), Poincaré plot standard deviation along the line of identity (SD2), and the ratio of SD1-to-SD2 (SD1/SD2), which have been defined as non-linear measures of HRV, were not included based on some findings of previous studies [11,21,22]. The authors noted that the inclusion of SD1 is not necessary when RMSSD is reported due to these two indices of HRV being mostly identical. Moreover, it has been previously indicated that SD2 and SDNN are equivalents and SD1/SD2 and LF/HF are strongly correlated; thus, reporting these parameters might be redundant [11,22]. Previous studies have confirmed that measurement of short-term HRV conducted via the Polar RS800CX heart rate monitor is a valid method for the assessment of a majority of HRV parameters [23]. Montaño et al. described a strong correlation between the results of the assessment of HRV indices using the Polar RS800CX and the results of HRV measures using an electrocardiogram [24]. Furthermore, it has been shown that the Polar RS800CX heart rate monitor exhibits a good test–retest reliability for the measurement of HRV [23].

### 2.4. Measurement of Arterial Stiffness

Data regarding arterial stiffness were collected non-invasively from the participants by computerized oscillometry using Cardio Vision MS-2000 (Osachi, Nagano, Japan). Systolic blood pressure (SBP), diastolic blood pressure (DBP), and pulse pressure (PP) were measured at the right brachial artery with the participants resting in a supine position. Measurement of the blood pressure with an oscillometric method was used to automatically calculate the arterial stiffness index (ASI). The measurement consisted of two recordings separately for 3 min, and the data from each recording were averaged. Measurement of ASI by non-invasive computerized oscillometry has been previously demonstrated to be a feasible method for an observation of the atherosclerotic changes in the vessels [25]. In addition, the measurement of ASI assessed by non-invasive computerized oscillometry has been previously demonstrated to be strongly correlated with the measurement of pulse wave velocity, which is a gold standard method for an evaluation of arterial stiffness and a predictor of cardiovascular disease and mortality [26].

### 2.5. Data Analysis

Mean and standard deviation (SD) were presented in the tables. The differences between the ASD and control groups in basic anthropometric characteristics, motor skills, HRV, and arterial stiffness were analyzed using the independent *t*-test. Additionally, the Shapiro–Wilk test was used to verify the normality of distribution for all variables. In case of the variables not following normal distribution, the Mann–Whitney U test was used to detect group differences. In particular, the differences between the groups in weight, BMI, fine motor integration, upper-limb coordination, bilateral coordination, balance, fine manual control, manual coordination, body coordination, mean NN, SDNN, RMSSD, logHF expressed in normal units, LF/HF, and SBP were analyzed via the Mann–Whitney U test. Statistical significance was set at *p* < 0.05 and the SPSS software (version 25.0) was applied for the statistical analysis. Effect sizes of the group differences were calculated using Hedge’s g, and the effect sizes were interpreted as small (0.2), medium (0.5), and large (0.8) [27].

## 3. Results

### 3.1. Basic Characteristics

Table 1 displays the basic characteristics of the participants. The ASD group was composed of 17 school-aged boys with ASD, while the control group consisted of 15 TD school-aged boys. The results did not show any statistically significant differences between the groups in age, height, and BMI. However, the results revealed a statistically significant difference between the groups in weight (ES = 0.76, *p* < 0.05, Table 1).

### 3.2. Comparison of Motor Skills between the Groups

Table 2 and Table 3 describe differences between the groups in motor skills assessed by the BOT-2. Table 2 presents the results of eight motor subtests, while Table 3 shows the results of motor composite scores.

Regarding the results of motor subtests, Table 2 displays the results of motor sub-tests expressed in scale scores. Children with ASD demonstrated statistically significant deficits in manual dexterity (ES = 0.96, *p* < 0.05), upper-limb coordination (ES = 0.91, *p* < 0.05), running speed and agility (ES = 0.82, *p* < 0.05), and strength (ES = 0.99, *p* < 0.05) compared to TD children (Table 2). On the other hand, the result of motor subtests did not indicate any statistically significant differences between the groups in fine motor precision, fine motor integration, bilateral coordination, and balance.

Regarding the results of motor composites, Table 3 lists the results of motor composites expressed in standard scores. Children with ASD displayed statistically significant deficits in manual coordination (ES = 0.97, *p* < 0.05), strength and agility (ES = 1.03, *p* < 0.05), and total motor composite (ES = 1.05, *p* < 0.05) compared to TD children (Table 3). However, the results did not show any statistically significant differences between children with ASD and TD children in fine manual control and body coordination (Table 3).

### 3.3. Comparison of Heart Rate Variability between the Groups

The differences between the groups in HRV are shown in Table 4. Regarding HRV time-domain measures, statistically significant differences between the groups were demonstrated in mean NN (ES = 0.85, *p* < 0.05), SDNN (ES = 0.92, *p* < 0.05), and RMSSD (ES = 0.85, *p* < 0.05). Furthermore, statistically significant differences between the groups were found in the HRV frequency-domain measures including log LF expressed in normalized units (ES = 1.07, *p* < 0.05), log HF expressed in normalized units (ES = 1.26, *p* < 0.05), log total power expressed in normalized units (ES = 0.79, *p* < 0.05), and LF/HF (ES = 1.70, *p* < 0.05). However, the results of the statistical analysis did not reveal any statistically significant differences in mean HR between children with ASD and TD children (Table 4).

### 3.4. Comparison of Arterial Stiffness between the Groups

The results of the statistical analysis of differences in arterial stiffness between children with ASD and TD children are shown in Table 5. The results did not indicate any statistically significant differences between the groups in SBP, DBP, PP, and ASI.

## 4. Discussion

The objective of the current study was to investigate the differences in motor skills, HRV, and arterial stiffness between children with ASD and TD children. The results of our study demonstrated that children with ASD had a significantly lower level of motor skills and significantly reduced HRV compared to TD children. On the other hand, the results did not demonstrate any statistically significant differences between children with ASD and TD children in arterial stiffness.

In the current study, children with ASD exhibited statistically significant deficits in manual coordination, strength and agility, and total motor composite compared to their TD counterparts. In agreement with our findings, Kaur et al. reported significantly reduced manual coordination and total motor composite in children with ASD in comparison with their TD counterparts [9]. Additionally, Kaur et al. also demonstrated deficits in fine manual control in children with ASD [9]. Nevertheless, the results of our study did not show such deficits. However, our study sample consisted solely of children from Taiwan and these children spend a considerable amount of time practicing their fine motor skills on a daily basis during activities, such as writing Chinese characters; therefore, this fact might partly explain the lack of statistical differences in fine manual control between the groups in our study. While the results of the present study demonstrated deficits in manual coordination, strength and agility, and total motor composite in children with ASD, a previous study by Odeh et al. has reported a significantly compromised performance of children with ASD in all five motor composites [5]. The authors of previously published studies suggested a variety of potential causes of motor deficits in children with ASD such as neuropathology associated with ASD [28], insufficient participation and engagement in daily physical activities [29], or the use of psychotropic medication [30].

Regarding motor subtests, the results of our study revealed statistically significant deficits in manual dexterity, running speed and agility, upper-limb coordination, and strength in children with ASD. In agreement with the results of our study, Odeh et al. have reported statistically significant deficiencies in motor subtests of manual dexterity, running speed and agility, upper-limb coordination, and strength in children with ASD [5]. In addition, Odeh et al. described that children with ASD achieve significantly lower scores in motor subtests of bilateral coordination and balance compared to TD children [5]. Nevertheless, the results of our study did not indicate any statistical differences between the groups in motor subtests of bilateral coordination and balance. It has been previously indicated that the motor skill development in children with ASD is inversely correlated with their symptoms severity [31,32]. The inconsistent findings regarding bilateral coordination and balance in children with ASD between Odeh et al. and our studies might be partly explained by different severity of ASD symptoms [5]. Our study recruited children with mild ASD, but their ASD participants demonstrated moderate to severe symptoms.

The results of our study confirmed that children with ASD exhibited a reduced resting HRV in comparison to TD children. Similarly, Thapa et al. and Lory et al. have reported decreased HRV in children with ASD compared to their TD counterparts [13,14]. In particular, Thapa et al. demonstrated statistically significant reductions in HRV parameters including RMSSD and HF power, which was in agreement with the results of our study [13]. On the contrary, Bricout et al. did not detect any differences between children with ASD and TD children in RMSSD [33]. The differences in resting HR between children with and without ASD has been noticed in numerous studies in recent years; however, some inconsistent findings have been reported. Some studies have demonstrated elevated resting HR in children with ASD [12,13,34], whereas other studies did not detect any differences in resting HR between children with ASD and TD children [33,35]. It has been suggested that higher resting HR signals increased sympathetic activity or impaired control of the parasympathetic system in children with ASD [36]. The results of the present study indicated a trend of elevated resting HR in children with ASD; however, possibly due to a smaller sample size, the results did not reach statistical significance. In addition, our results demonstrated that children with ASD exhibit significantly reduced SDNN values compared to TD children. In contrast, Thapa et al. did not identify any statistically significant differences in SDNN between children with and without ASD [13]. Furthermore, the results of our study showed significantly lower values of HF power in children with ASD; on the contrary, Tessier et al. did not find any significant differences in HF power between children with and without ASD [37]. A possible explanation for these disparities might be that Tessier et al. assessed HRV in their participants exclusively in the evening, which could possibly influence the findings [37].

In the present study, we did not identify any statistically significant differences in arterial stiffness between children with and without ASD. Heffernan et al. investigated arterial stiffness in children with ASD; however, the results of their study were not compared with a control group [18]. Nonetheless, Heffernan et al. used a previously published normative data of arterial stiffness in TD children for a comparison with the sample of children with ASD, and the authors concluded that the sample of children with ASD from their study were placed in the 95th percentile for arterial stiffness [18]. On the other hand, a study by Otsuki and Ohashi examined differences in arterial stiffness between physically active adults with and without ASD, and the results did not demonstrate any statistically significant differences between these two groups [38]. Moreover, Otsuki and Ohashi reported that physically active adult males with ASD displayed notably lower arterial stiffness compared to less physically active adult males without ASD [38]. These results might suggest that physical activity participation is probably the key factor determining potential differences in arterial stiffness between individuals with and without ASD. Sakuragi et al. focused on the relationship between physical activity and arterial stiffness in TD children and the authors demonstrated a statistically significant inverse relationship between physical activity and arterial stiffness [39]. However, this relationship has not been examined in children with ASD. The results of our study did not show any statistical differences in arterial stiffness between children with and without ASD. This could possibly be explained by similar physical activity participation in participants with and without ASD; however, the physical activity participation of our participants was not evaluated therefore, the results of our study cannot support this idea.

In the current study, we did not detect any statistically significant differences in SBP, DBP, and PP between children with and without ASD. In accordance with the results of our study, previous studies by Corbett et al. and Bricout et al. both reported that there were no statistically significant differences between children with and without ASD with respect to SBP and DBP [33,35]. Contrary to our findings, Ming et al. have reported significantly increased DBP in children with ASD compared to TD children [34]. Ming et al. noted that these results might possibly be explained by increased vasoconstriction caused by elevated sympathetic activity in children with ASD [34]. On the other hand, Otsuki and Ohashi examined differences in SBP and DBP between physically active adults with and without ASD, and the authors concluded that there were no differences between these two groups [38]. These findings might suggest a positive role for physical activity in managing blood pressure in individuals with ASD; however, it is difficult to compare these findings with the results of our study due to the differences in the studied populations.

The study design of our research paper presents a number of limitations which could possibly influence the interpretation of the findings. Firstly, the present study included a rather small sample size; therefore, it is possible that the results are not representative of the whole population of children with ASD and TD children. Secondly, the potential influence of medication use among the participants on the results of our study was not examined. Thirdly, the differences between the groups in HRV were evaluated by short-term HRV recording in the present study. Thus, the application of 24-h long-term HRV recording could be suggested to substantiate the results and further examine cardiac autonomic regulation in children with ASD. Lastly, the current study predominantly examined predominantly children with mild symptoms of ASD; therefore, this should be taken into consideration when comparing the results of our study with the results of studies examining children with moderate or severe symptoms of ASD.

## 5. Conclusions

In conclusion, the present study demonstrated the existence of motor skills deficits in children with ASD. Specifically, children with ASD underperformed in the areas of manual coordination and strength and agility. In addition, the current study presented additional evidence about atypical ANS functioning in children with ASD; they displayed reduced HRV, illustrated by reductions in a number of HRV parameters including mean NN, RMSSD, and LF/HF. On the other hand, the results of our study did not substantiate the presence of increased arterial stiffness in children with ASD. The findings of the present study suggest that it might be necessary to adequately stimulate the motor development of children with ASD in order to negate existing motor skills deficits in these children. Moreover, more research should be dedicated to inventing potential treatment methods for autonomic dysregulation in children with ASD in order to prevent possible adverse health consequences.

## Figures and Tables

**Table 1:** Basic characteristics.

Variables	ASD (*n* = 17)	TD (*n* = 15)	ES	*p* Value
Age (years)	11.1 ± 1.0	11.0 ± 0.5	0.08	0.833
Height (cm)	148.27 ± 8.88	143.29 ± 6.15	0.64	0.079
Weight (kg)	43.31 ± 11.76 *	36.85 ± 7.85	0.76	0.045
BMI (kg/m^2^)	19.48 ± 4.09	17.85 ± 2.95	0.49	0.180

Means ± SDs are presented for the variables. BMI: body mass index; ASD: autism spectrum disorder group; TD: typically developing group; ES: effect size. * *p* < 0.05, statistically significant differences between ASD and TD groups.

**Table 2:** Comparison of the BOT-2 motor subtests expressed in scale scores.

Variables	ASD (*n* = 17)	TD (*n* = 15)	ES	*p* Value
Fine Motor Precision	16.59 ± 5.08	17.73 ± 4.57	0.24	0.510
Fine Motor Integration	14.24 ± 5.90	17.27 ± 4.04	0.58	0.113
Manual Dexterity	14.12 ± 5.30 *	18.73 ± 4.13	0.96	0.011
Upper-Limb Coordination	11.71 ± 5.44 *	16.00 ± 5.01	0.91	0.018
Bilateral Coordination	14.24 ± 5.27	17.20 ± 3.28	0.59	0.093
Balance	15.18 ± 4.99	15.47 ± 4.03	0.13	0.704
Running Speed and Agility	14.47 ± 5.12 *	18.27 ± 3.95	0.82	0.027
Strength	11.12 ± 4.50 *	15.40 ± 4.14	0.99	0.009

Means ± SDs are presented for the variables. ASD: autism spectrum disorder group; TD: typically developing group; ES: effect size. * *p* < 0.05, statistically significant differences between ASD and TD groups.

**Table 3:** Comparison of the BOT-2 motor composites expressed in standard scores.

Variables	ASD (*n* = 17)	TD (*n* = 15)	ES	*p* Value
Fine Manual Control	50.35 ± 12.37	55.93 ± 8.70	0.52	0.156
Manual Coordination	44.47 ± 11.12 *	54.80 ± 10.08	0.97	0.010
Body Coordination	49.35 ± 10.45	53.47 ± 8.04	0.44	0.226
Strength and Agility	45.47 ± 9.23 *	55.07 ± 9.34	1.03	0.007
Total Motor Composite	46.71 ± 10.25 *	57.33 ± 9.93	1.05	0.006

Means ± SDs are presented for the variables. ASD: autism spectrum disorder group; TD: typically developing group; ES: effect size. * *p* < 0.05, statistically significant differences between ASD and TD groups.

**Table 4:** Comparison of heart rate variability at rest.

Variables	ASD (*n* = 17)	TD (*n* = 15)	ES	*p* Value
Mean HR (bpm)	92.88 ± 13.86	84.47 ± 11.76	0.65	0.076
Mean NN (ms)	650.48 ± 102.68 *	731.25 ± 102.29	0.85	0.027
SDNN	34.79 ± 17.80 *	56.45 ± 31.62	0.92	0.018
RMSSD	28.15 ± 23.05 *	55.74 ± 44.00	0.85	0.027
Log LF (nu)	1.22 ± 0.05 *	1.15 ± 0.08	1.07	0.004
Log HF (nu)	1.44 ± 0.06 *	1.54 ± 0.08	1.26	0.002
Log total power (nu)	1.65 ± 0.04 *	1.69 ± 0.06	0.79	0.034
LF/HF	0.68 ± 0.14 *	0.46 ± 0.13	1.70	<0.001

Means ± SDs are presented for the variables. ASD: autism spectrum disorder group; TD: typically developing group; ES: effect size; HR: heart rate; NN: normal-to-normal interval; SDNN: standard deviation of the NN intervals; RMSSD: root mean square of successive NN interval differences; LFnu: low-frequency power in normalized units; HFnu: high-frequency power in normalized units; LF/HF: the ratio of LF-to-HF power; log: log-transformed values.* *p* < 0.05, statistically significant differences between ASD and TD groups.

**Table 5:** Comparison of arterial stiffness at rest.

Variables	ASD (*n* = 17)	TD (*n* = 15)	ES	*p* Value
SBP (mmHg)	100.47 ± 11.16	101.23 ± 5.66	0.09	0.791
DBP (mmHg)	59.82 ± 6.94	57.97 ± 4.55	0.31	0.373
PP (mmHg)	41.41 ± 6.67	43.27 ± 5.75	0.30	0.409
ASI	45.35 ± 11.28	44.27 ± 7.63	0.11	0.755

Means ± SDs are presented for the variables. SBP: systolic blood pressure; DBP: diastolic blood pressure; PP: pulse pressure; ASI: arterial stiffness index; ASD: autism spectrum disorder group; TD: typically developing group; ES: effect size.

## Data Availability

The data presented in this study are available from the corresponding author on reasonable request.
